# Evaluation of extraction kits and RT-qPCR systems adapted to high-throughput platform for circulating miRNAs

**DOI:** 10.1038/srep09430

**Published:** 2015-03-24

**Authors:** Geok Wee Tan, Alan Soo Beng Khoo, Lu Ping Tan

**Affiliations:** 1 Molecular Pathology Unit, Cancer Research Centre, Institute for Medical Research, Jalan Pahang, 50588 Kuala Lumpur, Malaysia

## Abstract

MicroRNAs regulate gene expression at the post-transcriptional level. Differential expression of miRNAs can potentially be used as biomarkers for early diagnosis and prediction for outcomes. Failure in validation of miRNA profiles is often caused by variations in experimental parameters. In this study, the performance of five extraction kits and three RT-qPCR systems were evaluated using BioMark high-throughput platform and the effects of different experimental parameters on circulating miRNA levels were determined. Differences in the performance of extraction kits as well as varying accuracy, sensitivity and reproducibility in qPCR systems were observed. Normalisation of RT-qPCR data to spike-in controls can reduce extraction bias. However, the extent of correlation for different qPCR systems varies in different assays. At different time points, there was no significant fold change in eight of the plasma miRNAs that we evaluated. Higher level of miRNAs was detected in plasma as compared to serum of the same cohort. In summary, we demonstrated that high-throughput RT-qPCR with pre-amplification step had increased sensitivity and can be achieved with accuracy and high reproducibility through stringent experimental controls. The information provided here is useful for planning biomarker validation studies involving circulating miRNAs.

MicroRNAs (miRNAs) are small non-coding RNAs that regulate gene expression at the post-transcriptional level^1^. A single miRNA can target multiple genes and a gene can be regulated by multiple miRNAs^2^. It was shown that miRNAs have high stability in plasma/serum under compromised storage conditions such as room temperature and multiple freeze-thaw cycles^3^,^4^,^5^. In recent years, a plethora of publications had reported the utility of circulating miRNAs as biomarkers for disease classification and prognosis^3^,^6^,^7^,^8^,^9^.

While miRNA profiling using microarray and next-generation sequencing (NGS) technologies are becoming common, reverse transcription - quantitative polymerase chain reaction (RT-qPCR) technique is still considered as the gold standard to validate differentially expressed miRNAs^10^ because it is the most sensitive platform to detect low amount and/or cell-free miRNAs^11^. In a recent study, it was revealed that the average validation rate for differentially expressed miRNAs between any two platforms of microarray, RT-qPCR and NGS was only 54.6%^11^. This inevitably created a bottleneck for biomarker study, because to compensate for the poor validation rate, large number of differentially expressed miRNAs is required to be validated in large sample size. With the advancement of technology, several high-throughput RT-qPCR platforms have been developed to cater to the needs of performing tens to hundreds of miRNA assays for hundreds to few samples in one run^12^,^13^,^14^,^15^. However, little is known about the performance and correlation among these high-throughput RT-qPCR platforms, especially when different primer designs as well as detection chemistry were used.

It is now known that handling bias can occur during the extraction step^16^ and plasma miRNAs may be contaminated by cellular miRNAs as well as haemolysis^16^,^17^,^18^. The intrinsic variability of circulating miRNAs levels is often overlooked^16^ and may confound studies. To date, only a few groups reported the variations of circulating miRNAs levels in different physiological states: significantly higher plasma levels of miR-1292 and miR-1323 in normal females as compared to normal males^19^, serum miR-141 level increased in prostate cancer patients^3^ as well as during pregnancy^20^. Within the same individual, it is unclear if factors such as diet, fasting status and time of blood collection may cause variation in miRNAs levels^21^.

The aims of our study were to use a high-throughput platform to evaluate the performance of five extraction kits and three RT-qPCR systems as well as the effects of different experimental parameters on circulating miRNAs levels in a cohort of healthy individuals. Issues common in biomarker validation, including intra- and inter-platform variations (extraction kits and RT-qPCR systems) as well as intra-individuals variations (sampling time point and type) were investigated. MiRNAs selected for evaluation include common cancer-associated markers and miRNAs known to be abundant in blood.

## Results

### Evaluation of extraction kits by RT-qPCR results

**Figure 1:**
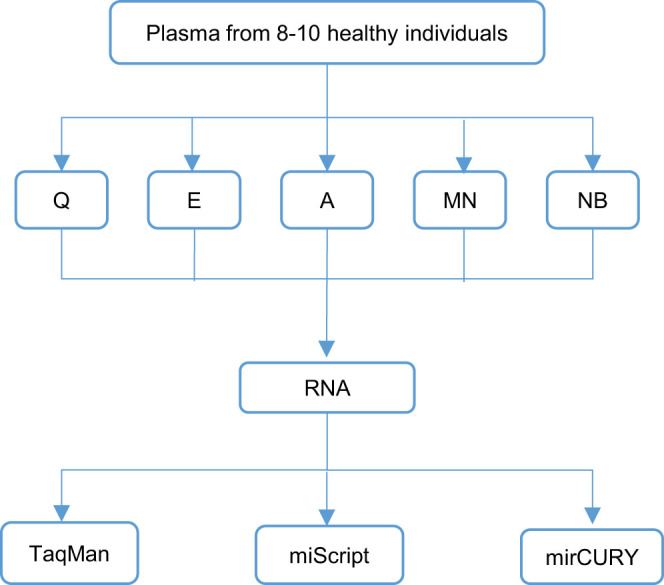
Overview of the experimental design for data depicted in Fig 2 to Fig 7. Plasma samples from eight to ten healthy individuals were aliquoted into five portions for isolation using five different extraction kits. The expression levels of 14 endogeneous miRNAs and two spike-in controls in these samples were evaluated using 3 RT-qPCR systems which were adapted to the high-throughput platform. Q, miRNeasy Serum/Plasma kit; E, miRCURY™ RNA Isolation Kit - Biofluids; A, mirVana™ PARIS™ Kit; MN, NucleoSpin® miRNA Plasma; NB, Plasma/Serum Circulating RNA Purification Kit.

**Figure 2:**
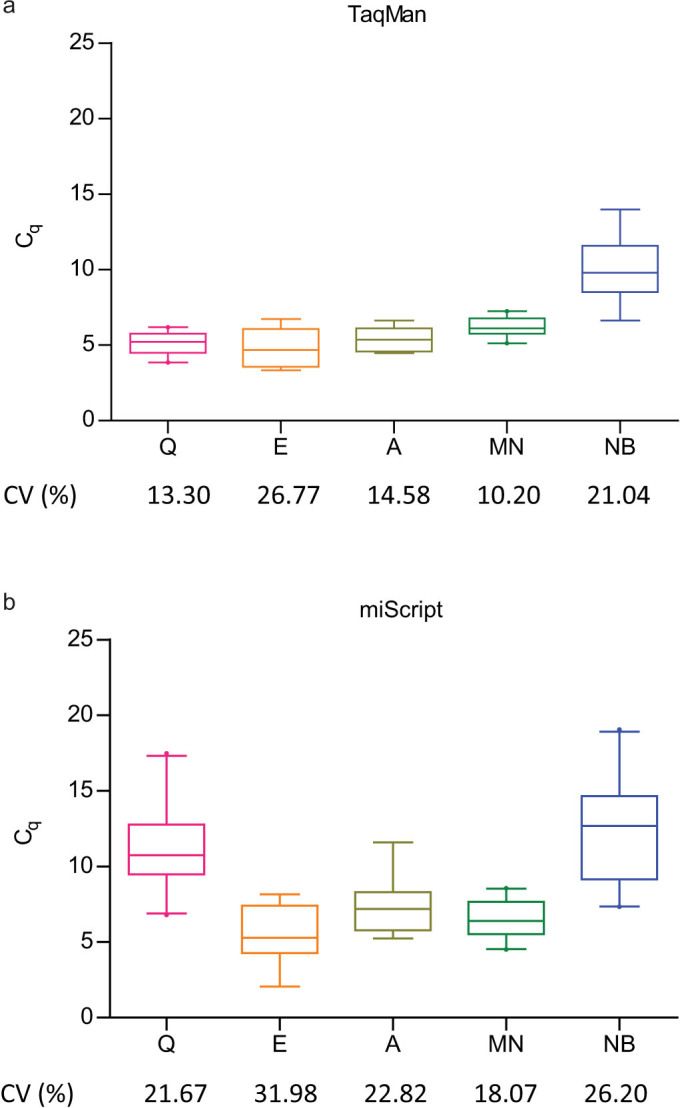
miRNA recovery of different extraction kits were evaluated by the C_q_ values for spike-in controls (cel-miR-39 and cel-miR-54) assayed in the (a) TaqMan and (b) miScript RT-qPCR system. Box plots with whiskers show 5–95 percentile of the C_q_ values for spike-in controls and the corresponding CVs are shown. The extent of extraction bias across samples was reflected in the range and CV of C_q_ values for spike-in controls. Higher median C_q_ compared to other extraction kits indicate poorer performance in miRNA recovery. Q, miRNeasy Serum/Plasma kit; E, miRCURY™ RNA Isolation Kit - Biofluids; A, mirVana™ PARIS™ Kit; MN, NucleoSpin® miRNA Plasma; NB, Plasma/Serum Circulating RNA Purification Kit.

Comparison of five extraction kits was done as illustrated in Figure 1. The performance of the kits were compared to each other by median and coefficient of variation (CV) of C_q_ values of the spike-in controls. Low C_q_ is an indication of high recovery while small CV would suggest less extraction bias across samples (Figure 2). When the TaqMan system was used for evaluation, we found that four different extraction kits were comparably good in recovering the spike-in controls, as evident by their similar low median C_q_ values (Figure 2a). In miScript system, good recovery was also observed in three extraction kits (Figure 2b). Extraction bias across samples was the least in NucleoSpin® miRNA Plasma (MN) kit, as it had the smallest CV of C_q_ values of the spike-in controls (Figure 2).

**Figure 3:**
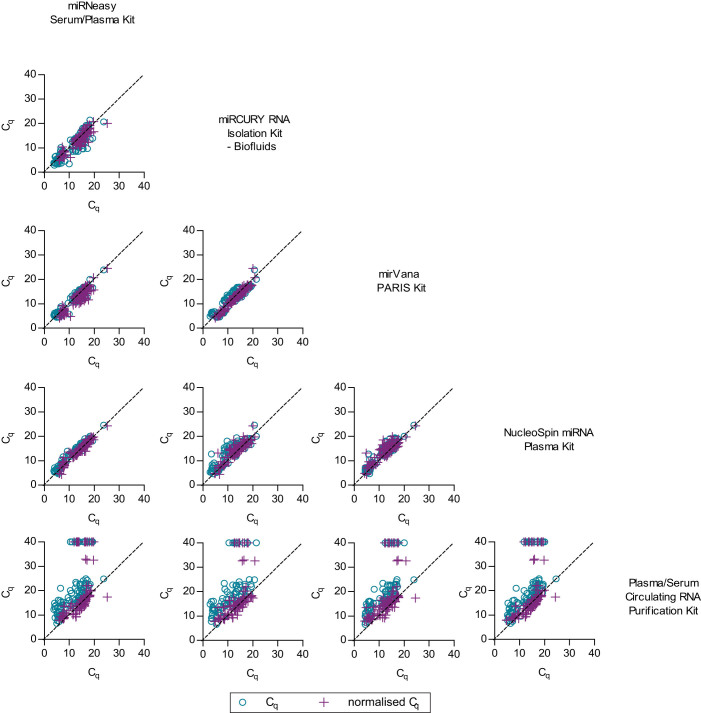
The effects of extraction kits on RT-qPCR results were evaluated using the TaqMan system. Pairwise comparison of extraction kits is shown in scatter plots. Each data point represents the average C_q_ value obtained from duplicate or triplicate wells of qPCR. Our results indicated that the correlation of data from same set of samples extracted using different kits can be improved by normalisation to spike-in controls.

During the evaluation of all data points including endogenous plasma miRNAs with the TaqMan system, it was noted that results from three extraction kits were highly correlated even before data was normalised to spike-in controls (Figure 3 and Supplementary Table S1). When normalisation was carried out, all extraction kits achieved significant high correlation, with intraclass correlation coefficient (ICC) values ranging from 0.811–0.991, p > 0.001 (Supplementary Table S1). Meanwhile, normalised miScript high-throughput RT-qPCR results from each kit had moderate agreement, with the ICC values ranging between 0.418–0.787 (Supplementary Figure S1 and Supplementary Table S2).

### Evaluation of RT-qPCR systems adapted to the high-throughput platform

**Figure 4:**
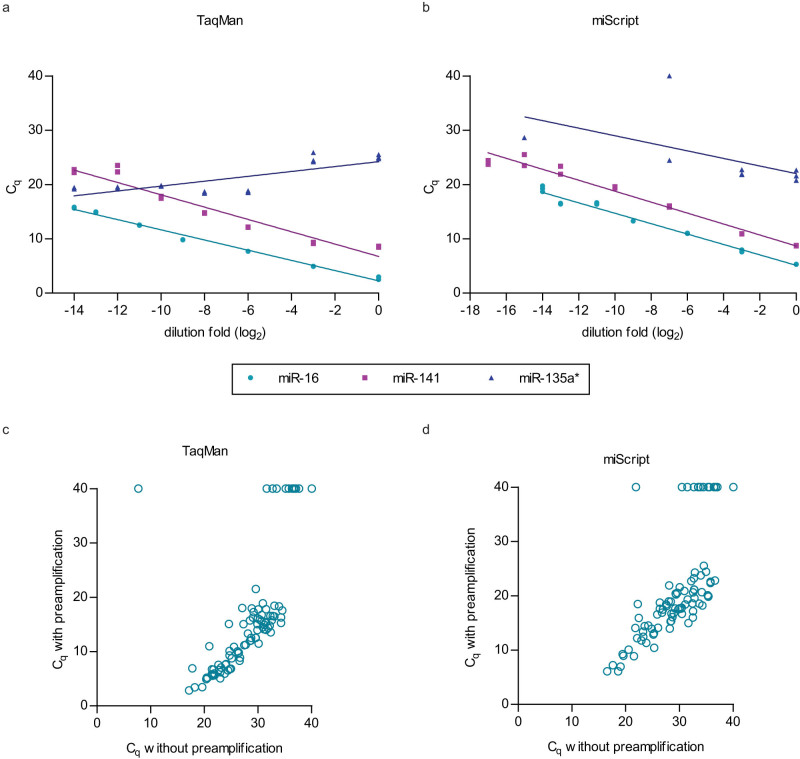
Examples of assays which showed linear amplification (miR-16 and miR-141) or non-linear amplification (miR-135a*) in serial dilution samples which were preamplified in the (a) TaqMan and (b) miScript systems. Significant correlation between the preamplified and non-preamplified data points was seen in (c) TaqMan (ICC = 0.888, p < 0.001) and (d) miScript (ICC = 0.862, p < 0.001).

In order to adapt the RT-qPCR systems to BioMark high-throughput platform, a preamplification step for RT products was carried out and stringent criteria for quality control were applied to rule out non-specific and non-linear amplifications. In our hands, after the preamplification step was introduced, all data points from the miRCURY system and some data points from the miScript system failed the melt-curve analysis and were omitted from further analysis (Supplementary Table S3 and representative graphs in Supplementary Figure S2). Linear amplification of serial diluted samples was not observed in miR-135a* assays of both miScript and TaqMan systems and therefore omitted from further analysis (Supplementary Table S3 and representative graphs in Figures 4a and 4b). Analysis of data which passed the quality control criteria indicated that in TaqMan as well as in miScript system, results of samples with and without preamplification were significantly correlated (ICC > 0.8, p < 0.001, Figures 4c and 4d).

**Figure 5:**
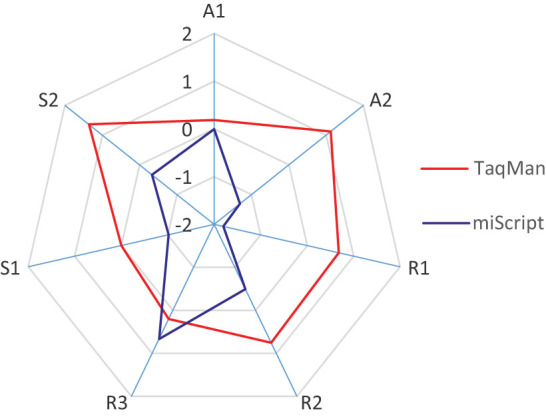
Comparison of TaqMan and miScript RT-qPCR systems adapted to the high-throughput platform by performance parameters which include reproducibility, accuracy and sensitivity. Transformation of data to z-score was carried out to facilitate direct comparison. A1, accuracy when measuring fold change of abundant miRNAs; A2, accuracy when measuring fold change of less abundant miRNAs, R1, reproducibility of RT replicates; R2, reproducibility of RT replicates after normalisation; R3, reproducibility of qPCR replicates; S1, sensitivity to detect the presence of plasma miRNAs and S2, sensitivity to detect the presence of miRNAs in titration points.

**Figure 6:**
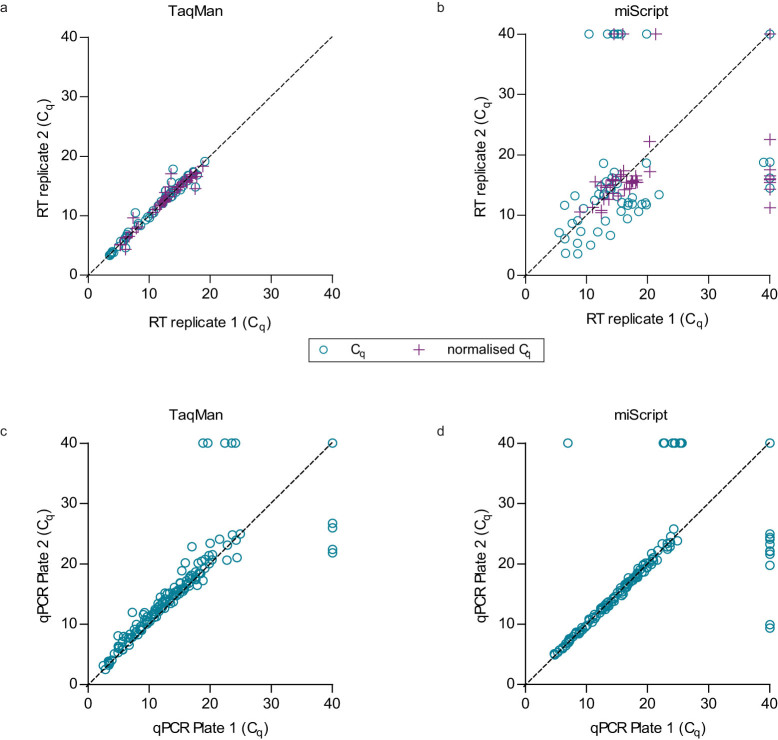
Evaluation of consistency between technical replicatesin RT-qPCR. Consistencies among (a) RT replicates in TaqMan system, (b) RT replicates in miScript system, (c) qPCR replicates in TaqMan system and (d) qPCR replicates in miScript system were evaluated by measuring the levels of 11–16 miRNAs in a same set of RNA samples (n = 5–14). Each data point in scatter plot represents the average C_q_ value obtained from the duplicate or triplicate wells of qPCR. Overall, significant correlations (ICC = 0.712–0.996, p < 0.001) between replicates were achieved when data were normalised to spike-in controls.

Reproducibility of the two RT-qPCR systems when adapted to the high-throughput platform was evaluated using data from RT replicates before and after data normalisation (R1 and R2) as well as data from qPCR replicates without normalisation (R3). Our results indicated that RT replicates in the miScript system had the lowest reproducibility score and could be moderately improved by data normalisation to spike-in controls (Figure 5). Overall, all technical replicates were highly correlated and achieved ICC > 0.9, except for RT replicates of miScript which had ICC of 0.712 after normalisation to spike-in controls (Figure 6 and Supplementary Table S4).

Accuracy of the two RT-qPCR systems was evaluated by data obtained from the standard curves (Supplementary Table S5). Accuracy of fold change for abundant miRNAs (A1, calculated from data point s1–s5) and accuracy of fold change when miRNA level was low (A2) in at least one of the samples (s6–s7) were determined. It was revealed that both RT-qPCR systems had equal accuracy in detecting fold change when miRNA levels were abundant but the accuracy for miScript decreased when miRNA levels were low (Figure 5).

**Figure 7:**
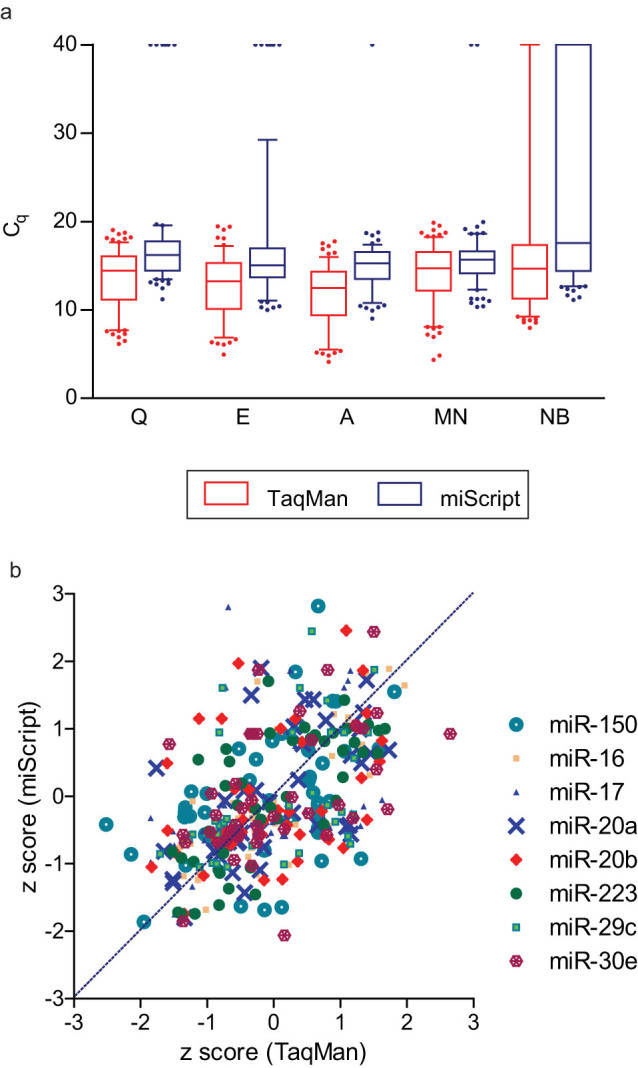
Comparison of different RT-qPCR systems adapted to the BioMark high-throughput platform. (a) Average C_q_ values for endogeneous plasma miRNAs were depicted in box plot with whiskers showing the 5–95 percentile. Irrespective of extraction kits being used, within the same set of samples, more undetermined C_q_ values were obtained in the miScript system. (b) Correlation of qPCR results from TaqMan and miScript systems may vary among different miRNA assays. Each data point represents the normalised C_q_ value transformed into z-score. Q, miRNeasy Serum/Plasma kit; E, miRCURY™ RNA Isolation Kit - Biofluids; A, mirVana™ PARIS™ Kit; MN, NucleoSpin® miRNA Plasma; NB, Plasma/Serum Circulating RNA Purification Kit.

Sensitivity of the two RT-qPCR systems was evaluated by the number of determined C_q_ values in plasma samples (S1) as well as in titration points (S2). Higher sensitivity scores were observed in the TaqMan system (Figure 5). Also, it was noted that, irrespective of extraction kits being used and within the same set of RNA samples, lower C_q_ range and less undetermined C_q_ values (C_q_ = 40) were obtained in the TaqMan system (Figure 2 and Figure 7a), indicating higher detection sensitivity for this system. When adapted to the high-throughput platform, results from both RT-qPCR systems were moderately comparable, with ICC values varying from 0.404–0.751 among different assays (Figure 7b).

### Evaluation of intra-individual variations in healthy cohort

**Figure 8:**
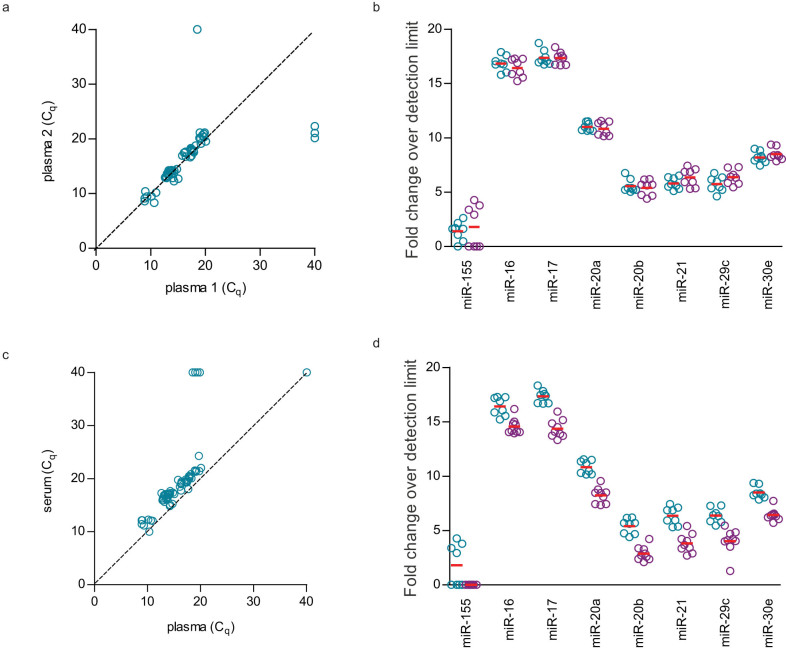
Intra-individual variations of eight circulating miRNAs were measured in nine individuals. (a) Significant correlation (ICC = 0.962, p < 0.001) and (b) no significant fold change (paired T test, p > 0.05) of circulating miRNAs was observed in plasma samples collected at different time points. In paired plasma and serum samples collected at the same time point, (c) significant correlation (ICC = 0.948, p < 0.001) and (d) significant fold change (paired T test, p < 0.05) of circulating miRNAs were observed.

Significant high correlation of plasma miRNA levels (ICC = 0.962, p < 0.001) was observed in blood samples collected at different time points from the same set of individuals (Figure 8a). There was no significant difference (Two-tailed paired T-test, p > 0.05) in plasma miRNA levels at different time points (Figure 8b).

Significant high correlation (ICC = 0.948, p < 0.001) was also observed between paired plasma and serum samples (Figure 8c). However, miRNA levels were significantly different (Two-tailed paired T-test, p < 0.05) between plasma and serum samples (Figure 8d).

## Discussion

For biomarker studies, it is now known that poor gene selection and validation strategies as well as technical biases including sampling errors, inconsistencies in sample processing and varying experimental parameters may lead to high prediction accuracy in the initial training set but low prediction accuracy in independent dataset^22^,^23^,^24^. Circulating miRNAs have recently emerged as a new class of potential biomarkers and many groups had reported the identification of circulating miRNAs as disease biomarkers using different platforms. In this study, different extraction kits and RT-qPCR systems adapted to a high-throughput platform for circulating miRNAs were compared. Performance parameters including miRNA recovery, reproducibility, accuracy, detection sensitivity and inter-platform correlation were evaluated. Also, the effects of sampling time and sample type on circulating miRNA levels within a cohort of healthy individuals were examined.

Performance of extraction kit is usually indicated by the purity and yield of extracted RNA measured in spectrophotometer. However, in the context of cell-free miRNAs, quantification of small RNAs using spectrophotometer or capillary electrophoresis can be affected by contamination of phenol^25^ and degradation of RNA^26^ while direct RT-qPCR results can be a better indicator for miRNA recovery. Benefiting from the advantage of high-throughput RT-qPCR platform, our study was able to evaluate larger number of technical replicates and miRNA assays compared to other studies^16^,^27^. Our findings suggested that among the five extraction kits that we tested, at least three of them were comparably good and poorer performance were seen in others (Figure 2). Surprisingly, albeit the differences in performance, normalisation to spike-in controls (Figure 3 and Supplementary Table S1) or by using a newer version of the extraction kit (Supplementary Table S6) greatly reduced the extraction bias and improved the correlation of RT-qPCR data. The use of synthetic miRNAs as exogeneous controls in circulating miRNA studies is in concurrence with Kroh et al^28^.

In this study, we evaluated three qPCR systems for their adaptation to the high-throughput platform which required preamplification of RT products. This preamplification step increases the sensitivity to detect less abundant miRNAs in low amount of samples but caution has to be made during analysis to exclude non-specific and non-linear amplifications. We used titration curve from pooled cell lines to estimate the limit of linear amplification and no-template controls to rule out false positive. It was observed that using our current protocol, we could not adapt the commonly used RT-qPCR system, miRCURY to the BioMark high-throughput platform. This is evidenced by its result seen in melt-curve analysis (routine step to examine non-specific amplification) in the preamplified samples (Supplementary Table S3). Discrepancies among qPCR systems are mainly due to their differences in the primer designs and chemistry of the qPCR reactions. While the TaqMan system uses probe for detection and design primers based on reference miRNA sequence, both miScript and miRCURY systems use DNA binding dye for detection and miScript have designed primers which can also detect isomiRs (miRNAs which show variation to the reference sequence). The combination of DNA binding dye and isomiR detection increase the difficulty of melt curve analysis, as it would be difficult for one to distinguish amplification of isomiRs from other non-specific amplification (Supplementary Figure S2). Compared to TaqMan, the miScript system produced more data points that had to be omitted due to its result in melt-curve analysis (Supplementary Figure S2). This may be one of the reasons leading to more undertermined C_q_ values and lower correlation observed in experiments carried out by the miScript system. Also, it is not surprising that lower detection sensitivity was observed in the miScript system, as the preamplification cycles we followed according to manufacturer's protocol were less for miScript as compared to TaqMan. It is likely that the miScript system will have improved detection sensitivity when protocol involving more pre-amplification cycles is optimised.

Varying circulating miRNA levels do not occur only in a diseased state, but sampling biases such as phlebotomy process^16^,^18^,^25^ and individuals' physiological state^30^,^29^ can also contribute to variation in circulating miRNA levels. Our results showed that within a group of healthy individuals, the levels of eight miRNAs in plasma samples collected at different time points were not significantly different. This include miR-223 and miR-16 which are highly abundant in blood cells^17^,^19^, suggesting that with the strict experimental control carried out in our study, haemolysis or cellular contamination was minimal. Also, within healthy individuals, plasma miRNA levels can be stable over time. However, owing to the small sample size and limited number of miRNAs evaluated in our time-point experiment, we cannot conclude that this would apply to all miRNAs. In concordance with the findings of McDonald et al^16^, our study also revealed lower miRNA levels in the serum compared to plasma from the same individuals. Lower levels of miRNAs will inevitably lead to problems of lower accuracy and lower detection rate.

In summary, our study had evaluated the performance of five extraction kits and two qPCR systems adapted to the high-throughput RT-qPCR platform for circulating miRNAs. Our results suggested that at least three extraction kits were comparably good in terms of recovery and consistency. Two qPCR systems successfully adapted to the high-throughput RT-qPCR platform had varying performance in terms of reproducibility, accuracy and sensitivity. Normalisation of RT-qPCR data to spike-in controls will greatly reduce the technical bias from extraction but not technical bias from the use of different RT-qPCR system. Lastly, increased detection sensitivity and high reproducibility of miRNA validation study using high-throughput RT-qPCR platform can be achieved by following stringent experimental parameters demonstrated in this study.

## Methods

### Samples collection

Ethics approval was obtained from the Medical Research & Ethics Committee (MREC), Ministry of Health Malaysia and methods were carried out according to the guidelines. Volunteer blood donors from the National Blood Blank and Institute for Medical Research were invited to donate blood samples for this study. Written informed consent was obtained from all 19 individuals prior to sample collection. All samples were collected from year 2012 to 2013. During each sampling, 5 ml of blood from healthy individual (no known critical disease at time of sampling) was collected in EDTA (for plasma) or SST tube (for serum). Blood sample was centrifuged at 2500 rpm for 10 minutes at room temperature. Plasma or serum was then transferred to a new tube and centrifuged at 3000 rpm for 10 minutes at room temperature. Samples were aliquoted into microcentrifuge tubes and then kept at −80°C prior to RNA extraction. All blood samples were processed within two hours after sampling. For the comparison of plasma collected at different time points, repeated blood samples were collected from the same patient at a gap of approximately one year. Sample characteristics are shown in Supplementary Table S7.

### RNA extraction

RNA from plasma samples were extracted using miRNeasy Serum/Plasma Kit (Qiagen, Hilden, Germany), miRCURY™ RNA Isolation Kit - Biofluids (Exiqon, Vedbaek, Denmark), mirVana™ PARIS™ Kit (Ambion, Texas, USA), NucleoSpin® miRNA Plasma (Macherey Nagel, Düren, Germany) and Plasma/Serum Circulating RNA Purification Kit (Slurry Format) (Norgen Biotek, Ontario, Canada). For optimal performance, all extractions were carried out according to manufacturer's protocol and both lysis buffer and isopropanol for the Plasma/Serum Circulating RNA Purification Kit (Slurry Format) were increased to 1 ml each. For paired plasma samples in time point experiment and the corresponding serum samples, automated RNA extraction was carried out using miRNeasy Serum/Plasma kit in QIAcube (Qiagen).

At the beginning of each extraction procedure, exogeneous controls cel-miR-39 and cel-miR-54 (Integrated DNA Technologies, Iowa, USA) were spiked into samples after the addition of lysis buffer. All eluted RNA samples were stored at −80°C until used. Details for comparison of extraction kits are indicated in Supplementary Table S8.

### Reverse transcription and quantitative real-time PCR (RT-qPCR)

High-throughput RT-qPCR was carried out on three different systems: TaqMan miRNA PCR system (Applied Biosystems, California, USA), miRCURY LNA microRNA PCR system (Exiqon) and miScript miRNA PCR system (Qiagen). The RT, preamplification and qPCR reactions were carried out according to previous publication^31^ and as indicated in Supplementary Table S8 and Supplementary Table S9. Nuclease free water was used as negative control. Samples from serial dilution of pooled synthetic miRNAs (Integrated DNA Technologies) and pooled cell line RNAs were used to construct standard curves and titration curves, respectively. All negative controls, serial diluted samples for standard curves and titration curves were included in all RT, preamplification and qPCR steps. All miRNA assays were performed in triplicate wells. High-throughput miRNA profiling was performed using the 48.48 or 96.96 Dynamic Array™ integrated fluidic circuits and run on the BioMark™ System (Fluidigm, California, USA). Correlation of preamplified samples with non-preamplified samples were also evaluated using six miRNA assays in the 96-well plate format and run on the ABI7500 FAST System (Applied Biosystems).

### Data analyses

Raw quantification cycle (C_q_) values were generated from Fluidigm Real-Time PCR Analysis. Wells with undetermined values were omitted from analysis. Average C_q_ was calculated from duplicate or triplicate wells using Microsoft Excel. Standard and titration curves were constructed from the C_q_ values of dilution points (Supplementary Table S5). Assays were excluded from the analysis when at least one of the following criteria was fulfilled: (1) non-linear amplification observed in the titration and/or standard curves, as demonstrated by coefficient of determination, R^2^ < 0.9, (2) undetermined C_q_ in more than 50% of the samples, (3) failure of melt curve analysis for the assays utilizing DNA-binding dye. Assays omitted from the analysis were listed in Supplementary Table S3.

As linear amplification can only be proven within the interpolation range constructed by titration or standard curve, samples with average C_q_ values beyond this range were considered as below detection limit and were assigned C_q_ value of 40.

Data normalisation was based on calculation as described below^28^:

For sample *X*,
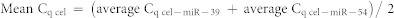

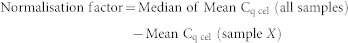

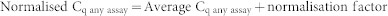


Reproducibility, accuracy and sensitivity were calculated as described below so that higher values would indicate better performance:
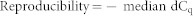

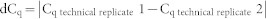

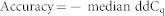


where ddC_q_ = | calculated (C_q_ standard point 1 - C_q_ standard point 2) - measured (C_q_ standard point 1 - C_q_ standard point 2) |
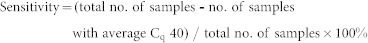


Fold change over detection limit in Figure 8 was calculated as indicated below^10^:

Fold change over detection limit = average C_q_ of highest detected value in linear amplification - normalised average C_q any assay_*.*

### Statistical Testing

In our experimental setting, ICC using two-way mixed model with absolute agreement was used as a measure for reproducibility when the same set of samples was processed and evaluated using the same experimental platform. In order to assess the correlation of data when different extraction kits, different RT-qPCR systems, different sampling time and type were used for the same set of samples, ICCs using two-way mixed model with consistency were calculated. For the computation of ICC in SPSS Statistics, only paired samples with determined values were included. Two-tailed paired T-test was used to evaluate the fold change of miRNA levels in plasma samples collected at different time points and in paired plasma and serum samples. All paired T-tests were calculated and graphs were plotted using GraphPad Prism.

## Author Contributions

G.W.T. designed the study, performed the experiments, analysed and interpreted the data and drafted the manuscript. A.S.B.K. provided comments on the design of study and reviewed the manuscript. L.P.T. designed the study, interpreted the data, and revised the manuscript. All authors have approved of the final version of the manuscript.

## Supplementary Material

Supplementary Information Supplementary Information
