# Supplementary material for: Evaluation of extraction kits and RT-qPCR systems adapted to high-throughput platform for circulating miRNAs
Source: Sci Rep. 2015 Mar 24;5:9430. doi: 10.1038/srep09430 (PMC4371102; doi:10.1038/srep09430)
Supplement: Supplementary Information [file srep09430-s1.pdf]

Evaluation of extraction kits and RT-qPCR systems adapted to high-throughput platform for circulating miRNAs.

Geok Wee Tan, Alan Soo Beng Khoo, Lu Ping Tan

Supplementary Table S1. ICC (two-way mixed model with consistency) of different extraction kits evaluated by the TaqMan system.[a]

	Q		E		A		MN	
	without normalisation	with normalisation	without normalisation	with normalisation	without normalisation	with normalisation	without normalisation	with normalisation
E	0.808***	0.892***						
A	0.927***	0.904***	0.875***	0.991***				
MN	0.972***	0.969***	0.784***	0.876***	0.905**	0.880***		
NB	0.708***	0.868***	0.602***	0.811***	0.760**	0.826**	0.722**	0.874***

Q, miRNeasy Serum/Plasma Kit; E, miRCURY™ RNA Isolation Kit - Biofluids; A, mirVana™ PARIS™ Kit; MN, NucleoSpin® miRNA Plasma; NB, Plasma/Serum Circulating RNA Purification Kit

[a]Data correspond to Figure 3

***p<0.001

**p<0.01

Supplementary Table S2. ICC (two-way mixed model with consistency) of different extraction kits evaluated by the miScript system.[a]

	Q		E		A		MN	
	**without normalisation**	**with normalisation**	**without normalisation**	**with normalisation**	**without normalisation**	**with normalisation**	**without normalisation**	**with normalisation**
E	0.463**	0.568***						
A	0.386**	0.463**	0.621***	0.733***				
MN	0.337**	0.439**	0.461**	0.657***	0.801***	0.787***		
NB	0.255[ns]	0.418**	0.293*	0.435**	NA	0.438**	0.456***	0.572***

Q, miRNeasy Serum/Plasma Kit; E, miRCURY™ RNA Isolation Kit - Biofluids; A, mirVana™ PARIS™ Kit; MN, NucleoSpin® miRNA Plasma; NB, Plasma/Serum Circulating RNA Purification Kit

[a]Data correspond to Supplementary Figure 1

***p<0.001

**p<0.01

*p<0.05

ns, not significant

NA, not available

Supplementary Table S3. Assays evaluated, analysed or excluded from analysis in different RT-qPCR systems adapted to the high-throughput platform.

miRNAs	RT-qPCR system			
	TaqMan[1]	miRCURY	miScript	TaqMan [2]
cel-miR-39	I	FMCA	I	I
cel-miR-54	I	FMCA	I	I
hsa-let-7a	I	FMCA	FMCA	NP
hsa-miR-135a*	NSA	FMCA	NSA	NSA
hsa-miR-141	NA	FMCA	NA	NP
hsa-miR-150	I	FMCA	I	NP
hsa-miR-155	NA	FMCA	I	I
hsa-miR-16	I	FMCA	I	I
hsa-miR-17	I	FMCA	I	I
hsa-miR-200a	NP	NP	NP	NA
hsa-miR-200b	NA	FMCA	NA	NP
hsa-miR-203	NP	NP	NP	NA
hsa-miR-20a	I	FMCA	I	I
hsa-miR-20b	I	FMCA	I	I
hsa-miR-21	NP	NP	NP	I
hsa-miR-223	I	FMCA	I	I
hsa-miR-29c	I	FMCA	I	I
hsa-miR-30e	I	FMCA	I	I
ebv-miR-BART11-3p	NP	NP	NP	NA
ebv-miR-BART14	NP	NP	NP	NA
ebv-miR-BART14#	NP	NP	NP	NA
ebv-miR-BART15	NP	NP	NP	NA
ebv-miR-BART1-5p	NP	NP	NP	NA
ebv-miR-BART16	NP	NP	NP	NA
ebv-miR-BART18-5p	NP	NP	NP	NA
ebv-miR-BART19-3p	NP	NP	NP	NA
ebv-miR-BART2	NP	NP	NP	NA
ebv-miR-BART22	NP	NP	NP	NA
ebv-miR-BART4	NP	NP	NP	NA
ebv-miR-BART5	NP	NP	NP	NA
ebv-miR-BART6	NP	NP	NP	NA
ebv-miR-BART6-5p	NP	NP	NP	NA
ebv-miR-BART7	NA	FMCA	NA	NA
ebv-miR-BART8	NP	NP	NP	NA
ebv-miR-BART8#	NP	NP	NP	NA
ebv-miR-BART9	NP	NP	NP	NA

[1]TaqMan assays performed for evaluation of extraction kits and RT-qPCR systems; [2]TaqMan assays performed for evaluation of plasma (collected at different time points) and serum; NP, not performed; I, Included in analysis; FMCA, Excluded in analysis due to fail melt-curve; NSA, Excluded in analysis due to non-linear amplification in positive controls or non-specific amplification in negative controls or both; NA, Excluded in analysis due to no amplification in >50% of samples.

Supplementary Table S4. Intraclass correlation coefficient (ICC) using two-way mixed model with absolute agreement for RT and qPCR replicates.

	**RT**		**qPCR**	
	**without normalisation**	**with normalisation**	**without normalisation**	**with normalisation**
TaqMan	0.970***	0.971***	0.977***	NP
miScript	0.078^{ns}	0.712***	0.996**	NP

***p<0.001

ns, not significant

NP, not performed.

Supplementary Table S5. Serial dilutions of standard curve and titration of pooled cell lines.

	Point	Dilution fold (log_2)	Concentration (g/µl)
**Standard Curve**	s1	0.0	2.5×10^{-12}
	s1a	-0.3	2.0×10^{-12}
	s2	-3.0	3.1×10^{-13}
	s3	-6.0	3.9×10^{-14}
	s4	-9.0	4.9×10^{-15}
	s5	-11.0	1.2×10^{-15}
	s6	-13.0	3.1×10^{-16}
	s7	-14.0	1.5×10^{-16}
**Pooled cell line RNA for TaqMan assays**	t1	0.0	2.0×10^{-7}
	t2	-3.0	2.5×10^{-8}
	t3	-6.0	3.1×10^{-9}
	t4	-8.0	7.8×10^{-10}
	t5	-10.0	2.0×10^{-10}
	t6	-12.0	4.9×10^{-11}
	t7	-14.0	1.2×10^{-11}
	PC1	0.0	2.0×10^{-7}
	PC2	-3.3	2.0×10^{-8}
	PC3	-6.6	2.0×10^{-9}
	PC4	-10.0	2.0×10^{-10}
	PC5	-13.3	2.0×10^{-11}
	PC6	-16.3	2.5×10^{-12}
	PC7	-18.3	6.3×10^{-13}
**Pooled cell line RNA for miScript assays**	t1	0.0	2×10^{-7}
	t2	-3.3	2×10^{-8}
	t3	-6.6	2×10^{-9}
	t4	-10.0	2.0×10^{-10}
	t5	-13.3	2.0×10^{-11}
	t6	-15.3	5.0×10^{-12}
	t7	-17.3	1.3×10^{-12}

t1-t7 were titration points used in all experiments except for PC1-PC7 which were used in experiments for Figure 5.

Supplementary Table S6. Comparison of new and old version of Plasma/Serum Circulating RNA Purification Kit (NB) extraction kits against all other extraction kits on TaqMan and miScript qPCR systems as shown by ICC.

	miScript				TaqMan			
	without normalisation		with normalisation		without normalisation		with normalisation	
	NB	NBR	NB	NBR	NB	NBR	NB	NBR
Q	0.589**	0.425*	0.382ns	0.498*	0.730***	0.839***	0.747***	0.828***
E	0.500**	0.674***	0.470*	0.662**	0.749***	0.865***	0.839***	0.933***
A	0.020ns	0.711***	0.540**	0.750***	0.760***	0.927***	0.840***	0.929***
MN	0.247ns	0.696***	0.352*	0.714***	0.764***	0.832***	0.830***	0.833***

Q, miRNeasy Serum/Plasma Kit; E, miRCURY™ RNA Isolation Kit - Biofluids; A, mirVana™ PARIS™ Kit; MN, NucleoSpin® miRNA Plasma; NB, Plasma/Serum Circulating RNA Purification Kit (older version); NBR, Plasma/Serum Circulating RNA Purification Kit (newer version).

***p<0.001
**p<0.01
*p<0.05
ns, not significant

Supplementary Table S7 . Donors characteristics and sample collection information.

Donors	Sample collection frequency		Duration between repeated collection	Age	Sex
	Plasma	Serum			
1	1	1	NA	44	M
2	1	1	NA	44	M
3	1	1	NA	43	M
4	1	1	NA	42	M
5	1	1	NA	53	M
6	1	1	NA	52	M
7	1	1	NA	42	M
8	1	1	NA	52	M
9	1	1	NA	42	M
10	1	1	NA	48	M
11	2	1	12 months	55	F
12	2	1	12 months	53	M
13	2	1	12 months	54	M
14	1	1	NA	57	M
15	2	1	12 months	47	F
16	2	1	13 months	57	F
17	2	1	13 months	55	F
18	2	1	13 months	55	F
19	1	1	NA	36	F

NA, not available

Supplementary Table S8. The starting input, spike-in, elution volume and correction factor for comparison among kits.

**Extraction kits**	**Plasma starting volume (µl)**	**Spike-in amount (fmol)**	**Elution volume (µl)**	**Correction factor for Cq**	
				**endogenous miRNAs**	**exogenous miRNAs**
miRNeasy Serum/Plasma Kit	200	12.5	25	0	0
miRCURY RNA Isolation Kit - Biofluids	200	25	50	-1	0
NucleoSpin miRNA Plasma	400	25	50	0	0
Plasma/Serum Circulating RNA Purification kit	400	25	50	0	0
mirVana PARIS Kit	400	25	100	-1	-1

Supplementary Table S9. Details of reverse transcription, preamplification and real-time PCR reaction mixes in all three systems.

Platform	Step		Reaction mix	Volume (ul)
**TaqMan**[a]	Reverse Transcription		100mM dNTPs	0.2
			RNase Inhibitor (20 U/µl)	0.15
			10x Reverse Transcription Buffer	1
			MultiScribeTM Reverse Transcriptase (50U/µl)	1
			RT primers pool[#]	3
			RNA sample	4.65
	Preamplification		TaqMan PreAmp Master Mix (2x)	5
			PreAmp Primer Pool[†]	2.5
			Diluted cDNA (1:4)	2.5
	Real-time PCR	Assay mix	TaqMan miRNA Assay (20X)	2.5
			Assay Loading Reagent (2X)	2.5
		Sample mix	TaqMan Universal Master Mix (2X)	2.5
			GE Sample Loading Reagent	0.25
			Preamp products (1:5)	2.25
**miRCURY** [b]	Reverse Transcription		5x Reaction buffer	2
			Nuclease –free water	5
			Enzyme mix	1
			RNA sample	2
	Preamplification		TaqMan PreAmp Master Mix (2x)	5
			PreAmp Primer Pool[‡]	2.5
			Diluted cDNA (1:10)	2.5
	Real-time PCR	Assay mix	miRCURY forward and reverse primers	2.5
			Assay Loading Reagent (2X)	2.5
		Sample mix	SsoFast EvaGreen Supermix with low ROX	2.5
			DNA Binding Dye Sample Loading Reagent	0.25
			Preamp products (1:10)	2.25
**miScript**[c]	Reverse Transcription		10x miScript Nucleics Mix	2
			5x miScript HiSpec Buffer	4
			miScript Reverse Transciptase Mix	2
			RNA sample	12
	Preamplification		5x miScript PreAMP Buffer	5
			HotStar Taq DNA Polymerase	2
			miScript Primer Assay Mix[§]	5
			Nuclease Free Water	7
			miScript PreAMP Universal Primer	1
			Diluted cDNA (1:5)	5
	Residual primers removal		Side Reaction Reducer	0.7
			Preamp products	17.5
	Real-time PCR	Assay mix	miScript primers	2.5
			Assay Loading Reagent (2X)	2.5
		Sample mix	SsoFast EvaGreen Supermix with low ROX	2.5
			DNA Binding Dye Sample Loading Reagent	0.25
			Preamp products (1:5)	2.25

[a]All reagents used are from Life Technologies, except Assay Loading Reagent and GE Sample Loading Reagent from Fluidigm.

[b]Reagents used for RT and all primers are from Exiqon, pre-amp master mix from Life Technologies, Assay Loading Reagent and DNA Binding Dye Sample Loading Reagent from Fluidigm and SsoFast EvaGreen Supermix with low ROX from BioRad.

[c]All reagents used are from Qiagen, except Assay Loading Reagent and DNA Binding Dye Sample Loading Reagent from Fluidigm and SsoFast

[#]RT Primer Pools consist of equal volume of 16 TaqMan RT primers (5X) diluted 1:2

[†]PreAmp Primer Pools consist of 16 TaqMan primers with a final concentration of 0.2 X

[‡]PreAmp Primer Pools consist of 16 miRCURY forward and reverse primers diluted 1:100

[§]miScript PreAmp Primer Mix consist of 16 miScript primers diluted to a final concentration of 0.4 X

Supplementary Table S10. Thermal cycling conditions for reverse transcription, pre-amplification, residual primers removal and real-time PCR.

Platforms	Steps	Cycles	Temperature	Time
**TaqMan**	Reverse Transcription	Hold	16°C	30 minutes
		Hold	42°C	30 minutes
		Hold	85°C	5 minutes
		Hold	4°C	∞
	Preamplification	Hold	95°C	10 minutes
		16 cycles	95°C	15 seconds
			60°C	4 minutes
	Real-time PCR	Hold	50°C	2 minutes
		Hold	95°C	10 minutes
		40 cycles	95°C	15 seconds
			60°C	1 minutes
**miRCURY**	Reverse Transcription	Hold	42°C	60 minutes
		Hold	95°C	5 minutes
		Hold	4°C	∞
	Preamplification	Hold	95°C	10 minutes
		16 cycles	95°C	15 seconds
			60°C	4 minutes
	Real-time PCR	Hold	50°C	2 minutes
		Hold	95°C	10 minutes
		40 cycles	95°C	15 seconds
			60°C	1 minutes
		Melt Curve Analysis	60°C	3 seconds
			60°C - 95°C	1°C /3 seconds
**miScript**	Reverse Transcription	Hold	37°C	60 minutes
		Hold	95°C	5 minutes
		Hold	4°C	∞
	Preamplification	Hold	95°C	15 minutes
		12 cycles	94°C	30 seconds
			60°C	3 minutes
	Residual primers removal	Hold	37°C	15 minutes
		Hold	95°C	5 minutes
	Real-time PCR	Hold	50°C	2 minutes
		Hold	95°C	10 minutes
		40 cycles	95°C	15 seconds
			60°C	1 minutes
		Melt Curve Analysis	60°C	3 seconds
			60°C - 95°C	1°C /3 seconds

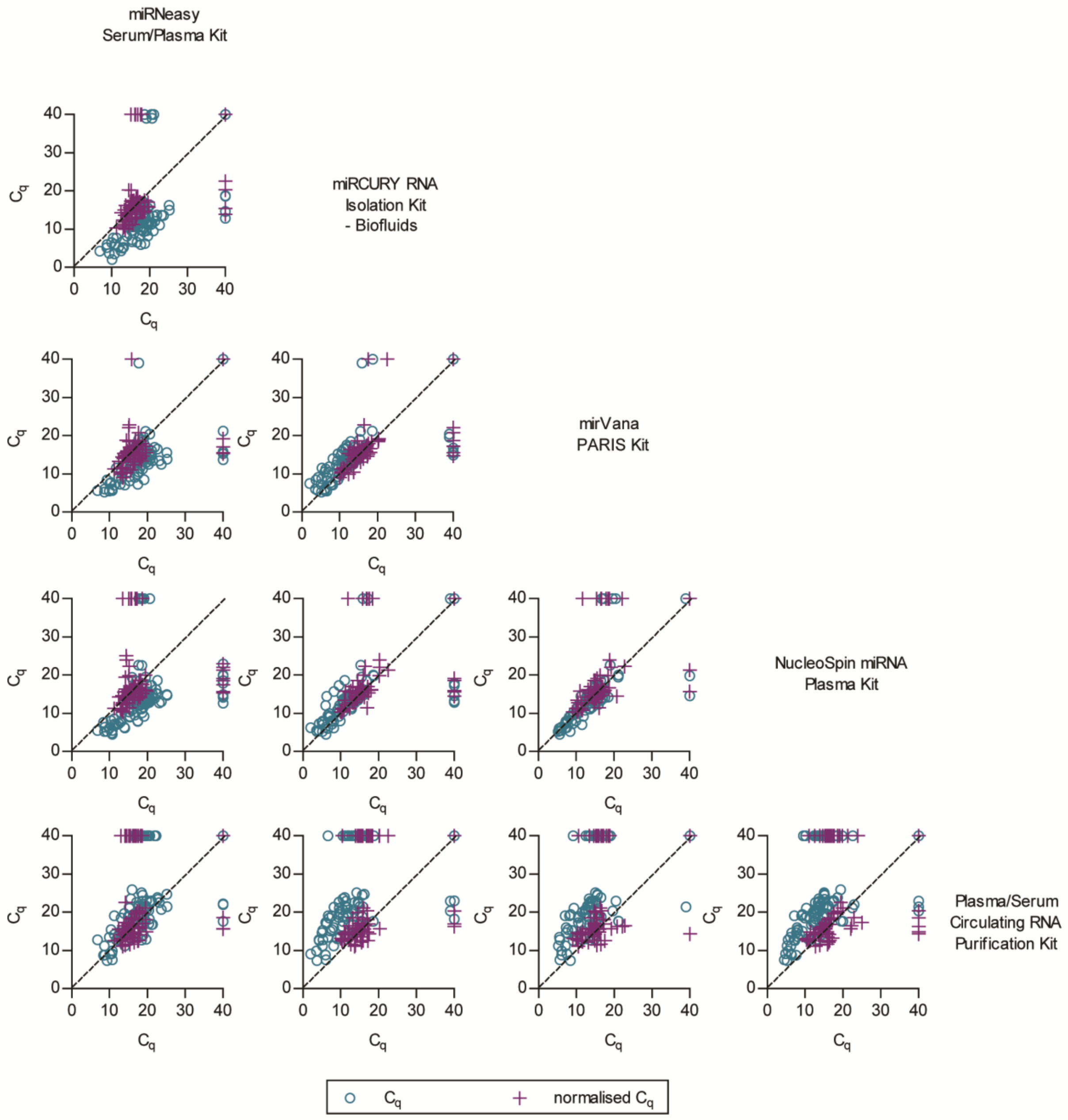

Supplementary Figure S1. The effects of extraction kits on RT-qPCR results were evaluated using the miScript system. Pairwise comparison of extraction kits is shown in scatter plots. Each data point represents the average C_q value obtained from duplicate or triplicate wells of qPCR. Our results indicated that the correlation of data from samples extracted using different kits can be improved by normalization to spike-in controls.

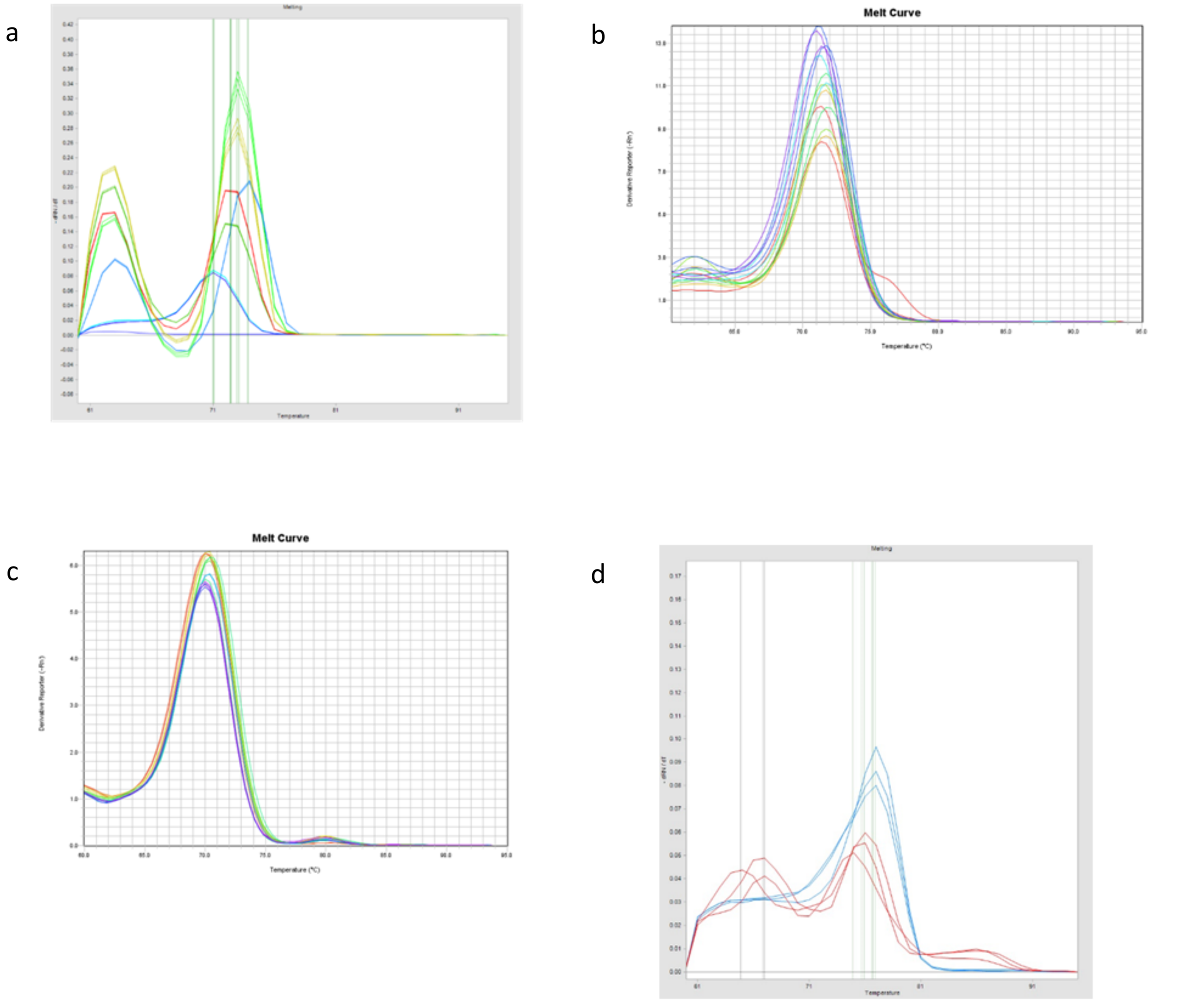

Supplementary Figure S2. Melt curves of RT-qPCR samples in different platforms. miRCURY's miR-16 was assayed in (**a**) preamplified samples, qPCR run on BioMark, Fluidigm (**b**) preamplified samples, qPCR run on ABI 7500 Fast Real Time PCR, and (**c**) non-preamplified samples, qPCR run on ABI 7500 Fast Real Time PCR. This assay failed quality control threshold when the preamplification step was applied and ran on BioMark, Fluidigm. (**d**) Melt curve of triplicates of two samples assayed for miScript's miR-141. One of the samples had fail-melt curve (red) and hence the C_q value assigned to 40.
